# Periodontal Endoscopy for Mechanical Debridement in the Non-Surgical Management of Peri-Implantitis: A Narrative Review

**DOI:** 10.3390/jcm14020346

**Published:** 2025-01-08

**Authors:** Sylwia Jakubowska, Bartłomiej Górski

**Affiliations:** Department of Periodontology and Oral Mucosa Diseases, Medical University of Warsaw, Binieckiego 6 St., 02-097 Warsaw, Poland; bgorski@wum.edu.pl

**Keywords:** periodontitis, periodontal diseases, peri-implantitis, peri-implant mucositis, peri-implant diseases, mechanical therapy, non-surgical periodontal treatment, periodontal endoscope, endoscope, endoscopy, perioscope

## Abstract

**Background**: The aim of the present narrative review is to synthesize the available scientific evidence on the effects of submarginal instrumentation with periodontal endoscopy and evaluate its’ potential efficacy in terms of the non-surgical therapy of peri-implantitis. **Methods**: The literature search was performed via electronic databases, including PubMed, Web of Science, Cochrane, and Scopus, and was supplemented by manual searching. A literature review was conducted addressing the following PICOS questions: (1) What is the efficacy of non-surgical submarginal instrumentation of the implant surface with the aid of a periodontal endoscope in patients with peri-implantitis? (2) What is the efficacy of non-surgical subgingival instrumentation performed with the aid of a periodontal endoscope compared with conventional subgingival instrumentation in patients with periodontitis, in terms of clinical parameters and patient-reported outcomes? Mechanical decontamination of the implant surface is crucial for resolving inflammation and arresting further bone loss. However, there is no consensus on the most effective treatment. Non-surgical therapy remains the initial intervention, focused on biofilm removal to control the disease, although with limited capability to achieve complete disease resolution as the presence of threads and the complex-structured implant surface hinder effective biofilm removal. This evokes the need for providing supporting technologies such as periodontal endoscopy, which enables direct visualization and precision, potentially enhancing the outcomes and reducing the necessity for surgical procedures and their associated risks. Within the limitations of this narrative review, periodontal endoscopy may offer a less tissue-invasive approach. Larger prospective studies and RCTs are needed to confirm these findings and guide clinicians in determining periodontal endoscopy’s suitability based on specific case characteristics.

## 1. Introduction

In recent decades, the prevalence of biological complications related to dental implants has risen correlating with the increasing adoption of implants as a solution for replacing missing or damaged teeth [1,2,3,4]. Even in cases of successful osseointegration, the loss of surrounding crestal bone can occur, induced by local inflammation.

A consensus report from the 2017 World Workshop on the Classification of Periodontal and Peri-Implant Diseases established criteria to improve diagnostic accuracy and provide clinical guidelines, yet variations in disease presentation continue to challenge consistency in diagnosis and treatment [5]. Peri-implant tissue health, peri-implant mucositis, and peri-implantitis represent a continuum. Changes are driven by inflammatory changes subsequent to microbial biofilm accumulation [6,7,8,9]. Peri-implant mucositis is an inflammatory lesion of the soft tissues surrounding an endosseous implant in the absence of the loss of supporting bone or continuing marginal bone loss. Peri-implantitis describes a plaque-associated pathological condition of implant-supporting tissues with signs of inflammation in the peri-implant mucosa and loss of the supporting bone [5,10].

Peri-implant diseases are highly prevalent and associated with significant morbidity [11]. A meta-analysis of 11 studies demonstrated a patient-level prevalence estimate of 43% for peri-implant mucositis and 22% for peri-implantitis [12,13]. Nowadays, peri-implantitis is considered to be the most difficult-to-manage biological complication associated with implants [3,14]. Hence, biological complications affecting osseointegrated implants have become a topic of major interest in contemporary dentistry [15].

This underscores the significance of identifying effective non-surgical interventions for peri-implantitis aimed at biofilm removal, which could contribute to a reduction in disease prevalence and the sustaining of long-term, healthy peri-implant tissues [16,17,18,19,20,21]. Although peri-implantitis management differs significantly from periodontitis, its treatment framework is largely based on stepwise strategies effective in periodontitis. Consequently, disease control should commence with less complex and minimally invasive methods, progressing to more advanced and invasive interventions as needed [22,23,24,25,26].

Until now, a variety of interventions, alone or in combination, have been investigated for the non-surgical treatment of peri-implantitis including mechanical [27], chemical (i.e., local or systemic antibiotics, and chlorhexidine irrigation), and light-mediated therapies (e.g., Er:YAG laser or photodynamic therapy) 1 [28,29,30]. Despite these various treatment strategies, the most effective treatment option for treating peri-implantitis lesions in a non-surgical way remains unclear [19,31,32,33,34,35,36,37,38,39,40].

Subgingival debridement performed without direct visual access has been reported to lack specificity, sensitivity, and reproducibility, making the complete removal of subgingival soft and hard deposits challenging [41,42,43,44,45]. In contrast, open periodontal flap surgery carries a higher risk of complications [46]. Exploring alternative approaches, supported by advancements in technologies such as PE (periodontal endoscope), is crucial for enhancing subgingival debridement outcomes [47].

Periodontal endoscopy is a minimally invasive technology that enables the visualization of subgingival tissues within a closed pocket through magnification and illumination. This technology facilitates the diagnosis and management of chronic periodontitis, peri-implant diseases, and other subgingival conditions. It is particularly beneficial for patients with probing depths exceeding 5 mm who are starting periodontal therapy or have not responded to conventional non-surgical debridement. Additionally, it supports periodontal maintenance in individuals with persistent inflammation, increasing probing depths, or residual pockets. It is also valuable for patients who are unwilling or unable to undergo surgical treatment due to medical, esthetic, or personal reasons [48,49,50,51]. Improved visualization of calculus, particularly in deeper pockets, complex bone defect anatomies, and intricate implant macrosurfaces, has the potential to enhance the effectiveness of non-surgical peri-implantitis therapy and minimize the need for surgical interventions. One approach to achieving this is the use of a periodontal endoscope, which has been reported to aid clinicians by providing enhanced visibility during treatment [52]. It is currently unknown if an additional use of periodontal endoscopy, in conjunction with sub-marginal instrumentation, can be of benefit in improving the outcomes of non-surgical peri-implantitis treatment. Therefore, within its limitations, the aim of the present narrative review is to synthesize the available scientific evidence on the effects of submarginal instrumentation with periodontal endoscopy and discuss its’ potential relevance in terms of non-surgical therapy of peri-implant diseases through a narrative review of the published literature.

## 2. Materials and Methods

Search strategy and data extraction: an electronic search of the literature was conducted in October 2024. Four databases: PubMed/Medline, Web of Science, Cochrane, and Scopus were screened for relevant articles. One reviewer (S.J) conducted the selection process according to the protocol discussed a priori with the second reviewer (B.G). The first reviewer imported all the articles from the databases into the Mendeley Reference Manager to identify and delete duplicates and screen the articles. Search terms were used for PubMed/Medline. The search strategy was customized according to the database being searched. The following strategy was used in the search: {(intervention) AND (outcome). (Intervention: [MeSH Terms] endoscopes OR [Text Word] perioscope OR perioscopy OR endoscopy OR endoscopic OR periodontal endoscope) AND (outcomes: [MeSH Terms] peri-implantitis OR periodontal diseases OR periodontitis OR non-surgical periodontal debridement OR periodontal therapy OR bleeding on probing OR clinical attachment loss OR probing depth)}.

The eligibility criteria of this narrative review were organized using the PICOS acronym.

P (Population): studies conducted in humans diagnosed with (a) periodontitis, (b) peri-implantitis/peri-implant mucositis, (c) in good general health, (d) and who did not undergo surgical or antimicrobial treatment.

I (Intervention): interventions for which the investigators (a) allocated the participants/teeth pairs/mouth-quadrants into test and control groups based on whether they underwent perioscopy-assisted SRP or conventional SRP, (b) used the perioscope to investigate the implant surface/tooth surface and surrounding inflamed tissues.

C (Comparison): (a) conventional SRP, (b) none when used as a diagnostic aid.

O (outcome measures): percentage of residual calculus, clinical measurements of PD, BOP, CAL, GI, PI, and RBL.

S (Study design): randomized clinical trials (RCT), cohort studies, case–control studies, case series, case reports, systematic reviews, and meta-analyses. All selected articles have to be in English.

Studies involving surgical or antimicrobial interventions, animal studies, in vitro studies, abstracts only, and narrative reviews were excluded.

Based upon the outlined PICOS criteria, the focused questions of this narrative review were as follows:

PICOS question 1: In patients with peri-implantitis, what is the efficacy of non-surgical submarginal instrumentation of the implant surface with the aid of a periodontal endoscope?

PICOS question 2: In patients with periodontitis, what is the efficacy of non-surgical subgingival instrumentation performed with the aid of a periodontal endoscope compared with conventional subgingival instrumentation in terms of clinical parameters and patient-reported outcomes?

Data from identified and relevant publications were extracted and, if indicated, presented in evidence tables and figures (flow chart). Overall findings were summarized in a narrative manner.

## 3. Results

**Figure 1:**
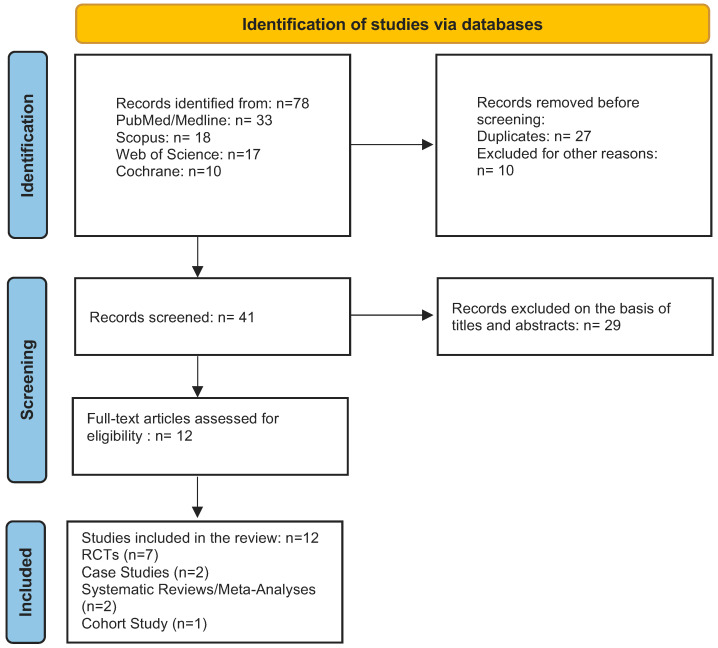
Flow chart of the selection process.

A total of 78 articles were obtained from the databases [Figure 1]. Subsequently, duplicates and articles that were not in accordance with our search strategy were removed. After revising the abstracts, 29 studies were discarded for their inappropriateness. After full-text reading, 12 studies investigating the implementation and effectiveness of the periodontal endoscope in peri-implant and periodontal diseases, non-surgical therapy, and diagnosis were evaluated [46,47,48,52,53,54,55]. The main characteristics of the selected studies and summary of reported outcomes are depicted in Table 1 (peri-implantitis/peri-implant mucositis), Table 2 (periodontitis), and Table 3 (systematic reviews/meta-analysis). Various possibilities of periodontal endoscope application and its relevance to peri-implant diseases were finally discussed referring to a priori determined PICOS questions 1 and 2.

### 3.1. PICOS Question 1

Among the studies included in the review, there were only two records identified that described the use of a periodontal endoscope in the treatment of peri-implant lesions. In 2009, Wilson TG [53] conducted a prospective study with the aim of exploring the relationship between excess dental cement and peri-implant disease using the dental endoscope. Excess dental cement was associated with signs of peri-implant disease in the majority (81%) of the cases. Clinical and endoscopic signs of peri-implant disease were absent in 74% of the test implants after the removal of excess cement. According to the author, the submucosal residues around the implant were readily identified with live endoscopic footage in a clinical setting. However, attempts to non-surgically dislodge the cement with piezoelectric and magnetostrictive mechanical devices while visualizing the cement with the dental endoscope were not successful in all cases and surgical access to three implants was necessary for complete cement removal. In 2016, Montevecchi et al. [54] published a case report describing the minimally invasive removal of the trapped material with the aid of the periodontal endoscope. The floss fibers trapped with the implant-prosthetic macrostructure correlated with the clinical and endoscopic signs of peri-implant inflammation. As reported by the author, the use of a perioscope was helpful for both diagnosis and treatment. A specific indication for this tool in this particular case derived from factors such as submarginal localization, small dimensions, and radio transparency of the residues. Therefore, the endoscope enabled direct, real-time visualization of the submarginal area with a minimally invasive approach, and it was possible to identify the foreign trapped body and remove it. According to the author, this approach was extremely welcomed by the patient and offered a better preservation of soft tissues with a potential benefit in the healing time and quality. Overall, the remission of inflammatory signs was observed after 10 days, and 3-month regular controls and debridements were scheduled. Re-examination after 1 year reported that probing depths returned to physiological values with the absence of bleeding, the marginal bone level remained stable, while the tissue contraction led to partial implant exposure.

### 3.2. PICOS Question 2

The umbrella review of systematic reviews and meta-analyses on the topic published in recent years recorded only two papers reviewing the efficacy of periodontal endoscopy on periodontitis therapy. In 2023, Ardila et al. [47] conducted a systematic review of three randomized clinical trials, which had a longer follow-up of at least 6 months (two RCTs) and 12 months (one RCT). Reviewed randomized controlled trials (RCTs) have demonstrated a statistically significant reduction in probing depth (PD) in the PEND group compared to controls at both 6 and 12 months of follow-up. The mean improvement in PD was 2.5 mm for the PEND group versus 1.8 mm for the control group (*p* < 0.05). Furthermore, after 12 months, the proportion of sites with PDs between 7 and 9 mm was markedly lower in the PEND group (0.5%) compared to the control group (1.84%) (*p* = 0.03). All RCTs consistently reported improvements in clinical attachment level (CAL), but only one found statistically significant differences. Additionally, reductions in bleeding on probing (BOP) significantly favored the PEND group, with an average decrease of 43% compared to 21% in the control group. Significant differences were also observed in the plaque index, favoring PEND. Earlier in 2017, Kuang et al. [55] conducted a systematic review and meta-analysis assessing eight RCTs. However, according to Ardila et al. [47] and the authors themselves, the reliability of the evidence is concerning due to the lenient selection criteria employed, which included clinical trials lacking follow-up data and studies with very small sample sizes. These methodological limitations introduce potential biases that warrant careful consideration. Nevertheless, the investigation of three studies reported advantages of perioscopy over traditional SRP in terms of BOP and GI. Investigation of four studies evaluated PD and found no statistical significance between periodontal endoscopy and traditional SRP. The authors were not able to perform a quantitative assessment of clinical parameters: BOP, GI, and PD. However, statistical analysis demonstrated that the percentage of residual calculus following perioscopy was significantly lower compared to scaling and root planing (SRP) alone, and that perioscopy was significantly more time consuming. Both authors strongly underscore the need for more RCTs with adequate sample sizes and longer follow-up periods to corroborate the current results. Only five RCTs with follow-up (range 4–12 months) and adequate sample sizes (20–38 patients), one case report, and two RCTs without follow-up were included in the scope of the narrative review based on their eligibility. Naicker et al. [61] attempted to determine if root surface debridement using perioscopy was more effective in improving clinical and radiographic parameters as compared to RSD. According to the authors, both test and control groups had significant improvements in clinical outcomes. After 12 months, the test group recorded a lower mean PD, BOP, PI, less change in gingival recession, and more radiographic bone gain, particularly around multirooted teeth. No difference was detected in CAL. Similar outcomes regarding the reduction in PD were observed by Wu et al. [48], since both treatments improved all clinical outcomes (PD, CAL, BOP, PI), although a greater decrease in PD and PI was notable in the perioscope group at both 3- and 6-month examinations. No differences between the groups were detected in CAL or BOP. The third RCT with a 4-month follow-up period conducted by Graetz et al. [56] reported the contrary results to the aforementioned studies. According to the author, CAL and PD improved in both groups during non-surgical treatment, while for BOP, no significant differences were found. However, higher PD reduction and CAL gain were correlated with the nPE group (control). Periodontal endoscopy was reported to be significantly more time consuming, but more surfaces with evident hard residues were detected with its aid. In the study by Wright et al. [52], when clinical parameters from all teeth were considered, the outcomes appeared very similar between the test and control groups. Interestingly, maxillary multirooted interproximal sites demonstrated a significant advantage with the use of the endoscope, as evidenced by a higher percentage of sites achieving clinical attachment level (CAL) gain, and mandibular multicoated interproximal sites favored conventional SRP in terms of CAL gain. Single-rooted teeth showed a significantly lower percentage of improved interproximal sites irrespective of the group assignment. Similar outcomes to Graetz et al. [56] were found by Blue et al., who reported that the adjunctive use of the perioscope was not found to be superior to traditional scaling and root planing with regard to pocket depth reduction and clinical attachment loss [59]. However, it enhanced the reduction in gingival inflammation and bleeding on probing. Michaud et al. [58] and Geisinger et al. [57] evaluated the percentage of residual calculus after traditional SRP and perioscope-assisted SRP by scanning the surfaces of extracted teeth with a stereomicroscope. Both studies revealed more residues at control versus test sites. According to Geisinger et al., at deeper probing depths, the use of the endoscope resulted in significantly less residual calculus; on the contrary, Michaud et al. reported no statistically significant differences between groups at deeper pockets or sites with furcation involvement. In the case report by Li et al. [60], a patient with stage 4, grade C periodontitis was treated with the aid of a periodontal endoscope as a part of a non-surgical multidisciplinary complex treatment. According to the author, the systematic and sequential NSPT provided satisfactory results and sustained periodontal health throughout the observation period.

## 4. Discussion

This review was focused on synthesizing the available scientific evidence on the effects of submarginal instrumentation with periodontal endoscopy and evaluating its’ potential efficacy in terms of non-surgical therapy of peri-implantitis. To the authors’ best knowledge, it is the first review to delve into this matter. Nevertheless, its level of evidence is troublesome, because there is a serious lack of reports on the application of periodontal endoscopy in peri-implant lesion treatment in the current literature [53,54]. On the contrary, perioscopy, in spite of being a relatively new technology, has been widely incorporated in periodontitis management with various results. The periodontal endoscope is designed to provide magnified, real-time visual access to subgingival areas, including periodontal pockets and peri-implant tissues. It consists of high-resolution glass fibers embedded in a plastic, disposable sheath, optic or digital camera with a magnification (typically ×24 to ×48), illumination, and irrigation system, allowing clinicians to visualize areas that are otherwise not visible during traditional periodontal therapy. The endoscope’s tip is gently inserted into the periodontal pocket or along the peri-implant tissues, ensuring minimal tissue trauma. The illuminated field and integrated irrigation ensure that even small deposits or defects are visible. The live footage is transmitted to a monitor, enabling the clinician to observe and assess the condition of the periodontal or peri-implant tissues while working [47,48,49,52,55,56,61].

Over the last few years, although many thorough and ceaseless studies have been conducted on therapeutic modalities, their predictability and effectiveness remain a controversial matter. Nevertheless, the EFP S3 clinical guidelines corroborate the statement that a stepwise approach mirroring the stages of a periodontitis treatment plan should be implemented [10,22]. Following the recommendations of the European Federation of Periodontology (EFP) S3 clinical guidelines, peri-implantitis therapy should start with a non-surgical step, re-evaluation, and, based on the results, progress to the surgical step or to SPIC [23,24,25,26].

Since peri-implant bone defects vary in configuration and severity, it is uncertain whether the tools for mechanical decontamination can access all areas of the implant surface for effective biofilm removal. The challenge lies in gaining proper access to implant surfaces, particularly in cases with deep peri-implant pockets and diverse implant surface designs. Poor implant surface accessibility, further complicated by three-dimensional microstructures, thread design, pronounced tapers in the implant shoulder area, and platform switching, makes complete removal of the biofilm almost impossible with the lack of direct visualization [41,42,43,44,45].

Given that non-surgical therapy faces inevitable limitations, management should be tailored to the clinical scenario and the patient’s needs specifically. Significant shortcomings include factors such as incomplete decontamination [16,41], a lack of visual access [47,62], unpredictable long-term outcomes [19], the impact on the implant surface (structure alteration, residual particles) [33,37,38], patient-related factors, and the dependence of adjunctive therapies. Additionally, limited hard tissue repair to increase peri-implant support and no gain in keratinized mucosa were observed [10]. Therefore, many alternative and adjunctive non-surgical approaches have been proposed and discussed recently such as antiseptics and local antibiotics, systemic antibiotics, air-polishing devices, lasers, or photodynamic therapy [23,24,28,63,64]. According to Faggion et al. [31], systematic review and meta-analysis combined treatments may obtain greater PD reduction, although with minimal differences compared with mechanical debridement alone. Similarly, the EFP S3 clinical consensus report [10] also suggests not to or recommends not to implement before-mentioned adjunctive modalities other than performing non-surgical supra- and sub-marginal instrumentation with curettes and/or sonic/ultrasonic devices. However, not only can some decontamination methods inadvertently alter the implant’s surface, affecting its biocompatibility and potentially impeding re-osseointegration, but also might leave behind abrasive particles or chemical residues, which could interfere with healing. Tran et al. [20] conducted an in vitro study investigating eight debridement protocols across three implant surfaces to assess both biofilm removal and surface alterations. Their conclusions underscore the fact that mechanical protocols for non-surgical debridement should be approached with caution, especially away from direct vision of the surface. This study showed the limitations of using traditional instrumentation, even when fitted with tips that are intended to be more “implant-safe”. The ultrasonic scaler with a titanium tip, carbon fiber, and titanium hand scalers and the Ni–Ti brush were, as a group, the least effective for biofilm elimination and caused gross surface alterations. In another study considering the limitations of non-surgical mechanical debridement, Steiger-Ronay et al. [45] developed an in vitro model of peri-implantitis to identify areas that are clinically difficult to clean by analyzing the pattern of residual stain after debridement with commonly employed instruments. Apically facing thread surfaces constituted the area with the most residual stain regardless of treatment approach. It may be probable that these non-accessed areas are the key to the implementation of more efficient instruments or techniques, which are aiming for the complete removal of pathogenic biofilm. Another rationale for attempting management with relatively simple approaches before escalating with treatment complexity and invasiveness is the improvement of the soft tissue conditions before surgical/corrective therapy or even avoiding the need for surgical procedures. In contrast, open periodontal flap surgery allows for the direct visualization of deposits, enabling more effective debridement. However, this approach may lead to postoperative soft tissue complications, root surface exposure, patient discomfort, and an extended healing period. Additionally, surgical therapy is more complex and carries a higher risk of complications in medically compromised patients, and some individuals may be reluctant to undergo surgical dental procedures. According to Heitz-Mayfield et al.’s systematic review [51], there is less morbidity of non-surgical compared to surgical therapeutic modalities, fewer sequelae compared to surgical therapy (particularly in the esthetic area), higher patient acceptability, and fewer postoperative complications. In the context of all the aforementioned research data, it is important to have alternatives that improve the efficacy and safety of submarginal debridement around dental implants with the support of new technologies such as periodontal endoscopy. It is a minimally invasive procedure that facilitates visualization of submarginal tissues, which increases the effectiveness of locating and eliminating residues on the tooth surface [48].

It might be presumed that the perioscope can visualize the hidden areas of the implant surface like the apically facing thread surface and significantly increase the precision of mechanical therapy, although there is no available evidence in the literature. However, authors evaluating calculus detection and removal with the aid of periodontal endoscopes in periodontal lesions report heterogeneous results. Geisinger et al. [57] recorded a statistically significant overall improvement in calculus removal with a perioscope, although without the knowledge of the clinical significance level of this improvement. Potential benefits were confirmed in a systematic review and meta-analysis by Kuang et al. [55]. Results by Michaud et al. [58] were contradictory. However, there are few randomized clinical trials comparing the relevant clinical parameters (PD, CAL, BOP, etc.) between perioscopy and SRP alone. According to a systematic review by Kuang et al., no sufficient evidence supports the advantages of perioscopy in clinical outcomes, whereas a systematic review by Ardila [47] favors periodontal endoscopy regarding clinical outcomes. No study was found that implemented the endoscope as an aid in the non-surgical stage of peri-implantitis treatment, comparing it with mechanical debridement alone. A prospective study by Wilson [53] describes using endoscopy to localize and remove cement remnants and records a remission of inflammation, but it does not provide any initial clinical parameters of inflamed tissues or longer follow-up, which might also provoke significant heterogeneity and bias. Montevecchi et al. [54] used the endoscope as a tool for magnifying the submarginal area to investigate the possible causes of tissue inflammation and remove the debris without the need for the surgical open flap approach. The author reports the success of the intervention, remission of inflammation, decrease in PD reduction, bone stability, and contraction of swollen tissues.

Based on the evidence gathered, regarding the use of perioscopy in periodontitis therapy and as a magnifying tool for diagnostics, the authors of the presented review can only speculate that it may provide advantages to conventional approaches. The authors observed during the research that the perioscope seemed particularly helpful as a diagnostic aid to investigate the submarginal area for cracks or fractures of teeth or implants or bone defect morphology; therefore, it may also be beneficial for choosing the appropriate treatment method or being a part of regular implant maintenance. According to meta-analytic estimates, individuals who adhere to regular maintenance programs exhibit approximately half the median prevalence of peri-implantitis compared to those who do not comply [3,14].

According to the reviewed literature and authors’ observations during the research, the potential indications for periodontal endoscopy in a non-surgical approach might be the following: before surgical corrective therapy, peri-implantitis with a horizontal defect and a swollen supra crestal component, infra osseous defects with mild severity, smokers (that excludes them from surgery), and mild-to-moderate peri-implantitis in the esthetic area [64]. However, the literature lacks evidence of PE use in such indications. Large, prospective, and longitudinal studies are necessary to evaluate the efficacy of this technology in these specific, clinical scenarios.

There are some crucial aspects and limitations that should be considered when interpreting available scientific data on the efficacy of the periodontal endoscope. Most of the studies were either nonrandomized, heterogeneous, or lacking longer observational periods and greater sample sizes, and there were a large number of case reports and a small number of case series, that could not provide the full information regarding the implantation procedure and possible risk of bias. More robust evidence could be gained from structured randomized clinical trials and multicenter studies. Another important issue is the fact that only a few studies mentioned the perioscope in diagnosing peri-implant lesions and no studies reported its use in peri-implant mucositis or peri-implantitis treatment in comparison to the conventional approach. Last but not least, the disadvantages that are adherent to the design of this narrative review (higher degree of bias) should be considered before drawing a conclusion.

## 5. Conclusions

Due to the scarcity of scientific evidence that met the inclusion criteria and the low certainty of the resultant evidence, no strong “evidence-based” conclusions can be drawn. Therefore, the authors indicate a possible area of interest and research for the future, in the scope of non-surgical treatment modalities for peri-implant diseases. In the authors’ opinion, there is not sufficient evidence to reject or corroborate the hypothesis that periodontal endoscopy can have a potentially beneficial influence on non-surgical peri-implantitis therapy. Whether the additional benefit of better visualization of residues during submarginal instrumentation provided by periodontal endoscopy is useful in particular clinical settings needs to be further and thoroughly studied.

## Figures and Tables

**Table 1:** Main characteristics of the selected studies on periodontal endoscope in peri-implant diseases treatment. PICOS Q#1.

Author Publication Date	Study Design	Diagnoses	Patients	Follow-Up	Intervention Control	Outcomes
Wilson T. G Jr 2009 [53]	Prospective cohort	Peri-implant disease	39 (20 females, 19 males, 41–78 years old) 42 test implants, 20 “control” implants (without signs of inflammation)	30 days	A dental endoscope was used to explore subgingivaly around the test and control implants. If excess cement was found, its presence was recorded and removed with hand scalers and piezoelectric mechanical devices with aid of the perioscope. In 3 cases, a flap approach was necessary to remove the cement thoroughly	Cement was associated with 34 of 42 test implants (80.95%) and with no control implants (0%). The clinical and endoscopic signs of peri-implant disease had resolved in 25 implants out of 33 test implants at the 1 month evaluation. No signs of peri- implant disease around control implants. The cause of the continued inflammation around the remaining 8 implants remained undetermined. There was no apparent relationship between the type of implant surface and the presence of inflammation or the retention of cement.
Montevecchi et al. 2016 [54]	Case report	Recurrent peri-implant mucositis	1 male, 66 years old	6 years	Intervention: Microbiologic test was performed. Anti inflammatory oral rinse was instructed twice a day. Submucosal investigation of implant surface with the aid of periodontal endoscopy. Filamentous foreign body was removed from implant surfaces under visualization of the endoscope.	At 10 days a complete remission was observed. At 1 year reevaluation, clinical stability and absence of any symptoms was observed. A microbiologic test showed the absence of periodontal pathogens. PD returned to accepted values, no BOP was detected. Tissue contraction led to partial implant exposure. 6 years later stability was confirmed in a clinical-radiographic evaluation.

**Table 2:** Main characteristics of the selected studies and summary of reported outcomes. PICOS Q#2.

Authors Publication Date	Study Design	Diagnoses	Patients	Follow-Up	Intervention CONTROL	Outcomes
Naicker et al. 2022 [46]	RCT	Moderate to severe chronic periodontitis.	38, 24 females/14 males. Mean age 52.	12 months	Test group: RSD with Perioscopy Control: RSD alone	Test group at 12 months: mean PPD 2.70 ± 0.2 mm Control at 12 months: mean PPD 2.98 ± 0.4 mm Test group at 3 and 12 months: %ofPD 7–9 mm 0.72 ± 1.2% and 0.5 ± 1% Control: 2.25 ± 2.9% and 1.84 ± 2.3%. At 0, 6,9,12 months: BOP and PI %lower in test group (*p* < 0.05) No differences in CAL between the groups. RBL gain for multi-rooted teeth: Test group: 0.83 +/ 0.5 mm. Control: 0.46 ± 0.4 mm
Wu et al. 2022 [48]	RCT	Moderate to severe chronic periodontitis. Stage 3 and 4.	37, 22 females/15 males. Mean age 37.	6 months	Test group: SRP with Perioscopy Control: SRP alone	Test group at 3 months: reduction in PD 3.45 ± 0.56 mm Control: 4.14 ± 0.59 mm Test group at 6 months: reduction in PD 3.12 ± 0.63 mm Control: 4.00 ± 0.68 (*p* = 0.001) No differences in CAL and BOP at 3 and 6 months.
Graetz et al. 2022 [56]	RCT, split-mouth study	Moderate to severe generalized periodontitis. Stage 3 and 4.	20, 10 females/10 males. Mean age 54	4 ± 1 month	Randomization into two quadrants for PE (test) or nPE (control) treatment.	At T1: CAL gain greater in nPE (*p* = 0.002), PD reduction higher in nPE (*p* = 0.038) Number of tooth surfaces with BOP: Lower in nPE (*p* = 0.026) TrT longer in PE group (*p* < 0.001). HDs detected in 14% sites in PE group and 6.2% in nPE group.
Geisinger et al. 2007 [57]	RCT, tooth pair	Min. 2 single-rooted teeth with a hopeless periodontal prognosis and min. 1 tooth with PD >/= 5 mm	15 patients, 50 pairs of teeth (100 teeth). 6 males/9 females. Age range 40–73.	No	Randomization in pairs of teeth. SRP with Perioscopy (test) Or SRP alone (control)	The difference between percentage of residual calculus on test and control surfaces, 2.14–3.13%, was statistically significant (*p* < 0.001) The percentage of residual calculus for PD < 6 mm at interproximal surfaces was 18.02–4.22% for control teeth and 16.90–3.39% for test teeth (*p* > 0.15). The percentage of residual calculus for PD > 6 mm at interproximal surfaces was 20.97–4.60% for control teeth and 16.83–3.95% for test teeth, and the difference was statistically significant (*p* < 0.001)
Wright et al. 2023 [52]	RCT, split-mouth study	Generalized periodontitis. Stage 2 or 3.	25, 36% male, 64% female. Mean age 42.7 years.	12 months	Randomization into two quadrants for PE SRP (test) or nPE SRP (control) treatment.	Single-rooted teeth interproximal sites displayed a significantly lower percentage of improved sites (*p* < 0.05) than multirooted teeth for PD and CAL. Maxillary multirooted interproximal sites favored the use of the periodontal endoscope at the 3- and 6-months (*p* = 0.017 and 0.019, respectively) in terms of the percentage of sites with improved CAL (10% more). Mandibular multirooted interproximal sites showed more sites with improved CAL (10% more) using conventional SRP than with the periodontal endoscope (*p* < 0.05) at 1, 2, 3, 12 months. The difference between single-rooted teeth and multirooted teeth was less for the facial/lingual surface sites with no significant difference between those treated with PE or nPE (<0.05).
Michaud et al. 2007 [58]	RCT, tooth pair	Stage/Grade not specified. At least two multi-rooted first or second molars with non-fused roots having a hopeless periodontal or restorative prognosis and at least one site with a PD >/= 5 mm	24 patients, 35 tooth pairs (70 teeth total)	No	Each tooth per pair was randomly assigned to receive endoscopy-aided SRP (test) or SRP alone (control).	Percentage of residual calculus: A statistically significant difference (*p* < 0.001) was observed only for mesial sur- faces (test: 10.93 ± 4.96 control: 14.33 ± 5.10) For interproximal surfaces, the difference of 2.63% was statistically significant (*p* = 0.003) (Table 1). For facial/lingual surfaces, the difference of 0.36% was not statistically significant (*p* = 0.652) No statistically significant differences in residual calculus displayed between groups at deeper probing depths or at sites with deep furcation invasions. At shallower interproximal sites with probing depths < 6 mm was significantly less residual calculus seen in roots treated with endoscopy (*p* = 0.020)
Blue et al. 2013 [59]	RCT, split-mouth study	Chronic, moderate periodontitis	26, 7 females, 19 males. Age 20–29 (5), 30–39 (3), 40–49 (6), 50–59 (9), 60+ (3)	3 months	Randomization into two quadrants for PE SRP (test) or nPE SRP (control) treatment.	No statistically significant differences in PD reduction and CAL gain: Mean PD reduced from 5.29 mm (±0.4) to 3.86 mm (±0.6) at visit 1 and to 3.55 mm (±0.8) at visit 2 in the test sites. In the control sites mean PD reduced from 5.39 mm (±0.5) to 3.91 at visit 1 and to 3.83 mm (±1.2) at visit 2. No difference in BOP between control and test during follow up. Mean change in BOP from baseline to visit 2 was greater for test sites (*p* = 0.036)
Li et al. 2022 [60]	Case report	stage IV/grade C periodontitis, Angle class I neutroclusion, dentition defects.	47 years old female	38 months total treatment period + 6 months follow up after treatment	No control	12 weeks after non- surgical periodontal therapy with a perioscope: BOP+ sites reduced from 86–39%. PI decreased from 100–17%. PD reduction in pockets > 5 mm: From 63% to 2%.

**Table 3:** Tabular presentation of quantitative and qualitative findings of an umbrella review. PICOS Q#2.

Authors Publication Date	Number of Included Studies	Studies Qualification	Selection Criteria	Results
Ardila et al. 2023 [47]	3	PICOS: Population: Patients diagnosed with periodontitis without the presence of systemic diseases. Intervention: PEND during subgingival debridement. Comparison: subgingival debridement. Outcomes: primary, probing depth, and clinical attachment level; secondary, bleeding on probing. Study design and follow-up: randomized clinical trials with follow-up of at least 6 months.	Inclusion criteria: RCT with >6 months follow up, patients diagnosed with periodontitis who were systemically healthy and treated with subgingival debridement and PEND. Only sample size > 30 patients. Exclusion criteria: Surgical or antimicrobial interventions, in vitro assays, animal studies, duplicate investigations	All 3 RCTs found a greater reduction in probing depth in the test group compared to the controls (*p* < 0.05) Probing depth reduction was 2.5 mm for PEND and 1.8 mm for the control groups, respectively (*p* < 0.05). 1 RCT described that the PEND group presented a significantly inferior proportion of probing depths of 7 to 9 mm at 12 months (0.5%) as compared to the control group (1.84%) (*p* = 0.03). 1 RCT described statistically significant differences in CAL gain after 6 months, with an improvement of 1.73 mm and 1.13 mm for the PEND and control groups, respectively (*p* < 0.001) 2 RCTs described significant differences in BOP, with an average reduction of 43% in test groups versus 21% in the control groups.
Kuang et al. 2017 [55]	8	Focused question: On the basis of the RCTs included in this study, what were the effects of using periodontal endoscopy after periodontal therapy on the practitioner’s ability to remove calculus, the average length of treatment time and the clinical parameters?	Inclusion criteria: RCTs, good general health, diagnosed with periodontitis, allocation of participants into test groups or control groups, outcomes including percentage of residual calculus, average treatment time, clinical measurements of BOP, GI, PD. English language. Exclusion criteria: Surgical interventions, participants with systemic diseases	The percentage of residual calculus after PEND was significantly less in comparison to traditional SRP (*p* = 0.002) (mean difference −3.18%) PEND took significantly more time than traditional SRP (*p* < 0.00001) (mean difference 6.01 min). 4 RCT studies analyzing PD described no difference between test and control groups. 3 RCT studies reported results on BOP and GI describing some advances of PEND over traditional SRP.

## Data Availability

Not applicable.

## Abbreviation

SPIC: supportive peri-implant care. SR: systematic review. PD: probing depth. PEND: periodontal endoscopy. CAL: clinical attachment level. BOP: bleeding on probing. GI: gingival inflammation. SRP: scaling and root planing. RSD: root surface debridement. PI: plaque index. NSPT: non-surgical periodontal treatment.
